# Artificial intelligence in total knee arthroplasty: clinical applications and implications

**DOI:** 10.1186/s43019-025-00295-0

**Published:** 2025-10-14

**Authors:** Kyeong Baek Kim, Gi Beom Kim, Jun-Ho Kim, Sang-Min Lee

**Affiliations:** 1 Department of Orthopedic Surgery, Pusan National University Yangsan Hospital, Research Institute for Convergence of Biomedical Science and Technology, 20, Geumo-ro, Mulgeum-eup, Yangsan-si, Gyeongsangnam-do, Yangsan, Republic of Korea; 2 Pusan National University School of Medicine, 20, Geumo-ro, Mulgeum-eup, Yangsan-si, Gyeongsangnam-do, Yangsan, Republic of Korea; 3 Shiley Center for Orthopaedic Research and Education at Scripps Clinic, Department of Molecular Medicine, Scripps Research, La Jolla, CA USA; 4 Department of Orthopedic Surgery, Yeungnam University Medical Center, Yeungnam University College of Medicine, Daegu, Korea; 5 Department of Orthopedic Surgery, Hallym University Sacred Heart Hospital. Seoul, Anyang, Republic of Korea

**Keywords:** Artificial intelligence, Deep learning, Total knee arthroplasty, Machine learning, Augmented reality, Virtual reality, Orthopedic

## Abstract

**Background:**

Artificial intelligence (AI), including machine learning (ML) and deep learning (DL), is increasingly being integrated into total knee arthroplasty (TKA) to improve accuracy, efficiency, and personalized care. These technologies enable the analysis of large, complex datasets to support evidence-based clinical decision-making across all phases of the surgical process.

**Main body:**

AI has demonstrated utility in multiple stages of TKA. In patient selection, ML algorithms can predict postoperative complications such as transfusion needs with high accuracy (AUC up to 0.842). For preoperative planning, DL techniques facilitate 3D anatomical reconstruction and implant size prediction, with some models achieving over 90% accuracy for exact component sizing, significantly outperforming traditional 2D templating. Intraoperatively, AI-assisted robotic systems and sensor technologies offer real-time feedback on alignment and soft tissue balancing. Postoperatively, AI-integrated wearable devices and mobile applications enable continuous monitoring and tailored rehabilitation; in some randomized trials, these tools have been associated with a statistically significant reduction in hospital readmission rates. Despite these advances, significant challenges remain, including algorithmic bias, a lack of model generalizability and explainability, and unresolved ethical and regulatory hurdles that present formidable barriers to widespread clinical implementation.

**Conclusions:**

AI has the potential to significantly reshape TKA by enabling more precise, data-driven, and patient-centered care. However, its promise is contingent on overcoming critical limitations. Broader implementation requires robust multicenter validation to ensure model reliability, the development of explainable algorithms to build clinical trust, and a commitment to responsible innovation. With continued progress, AI can serve as a powerful complementary tool to augment surgical expertise and enhance patient outcomes in orthopedic surgery.

## Background

The integration of artificial intelligence (AI) and deep learning (DL) technologies into orthopedic surgery is accelerating rapidly, with growing attention to their potential applications in total knee arthroplasty (TKA). AI supports clinicians by analyzing large-scale data and identifying complex patterns, thereby enabling more accurate and efficient decision-making. In orthopedic surgery, AI is expected to play a pivotal role in personalized diagnosis, surgical planning, intraoperative execution, and postoperative outcome prediction. Traditionally, surgical decision-making has relied heavily on the surgeon’s experience and intuition. However, with the advent of AI-based data analysis and predictive modeling, clinical decision-making is evolving into a more objective, data-driven, and quantifiable process [1–3].

AI refers to a broad field within computer science focused on developing technologies that mimic human cognitive functions such as learning, reasoning, and decision-making [4]. It encompasses both virtual elements, such as computing systems, and physical elements, such as robotics. Machine learning (ML), a subset of AI, employs algorithms that can learn from structured, preprocessed data with minimal explicit programming, enabling systems to automatically improve their performance through iterative training [5]. ML algorithms identify patterns within vast datasets and apply these insights to make predictions and decisions. Conversely, DL, a more advanced and specialized subfield of ML, uses multilayered artificial neural networks that mimic the neural connections in the human brain [6]. DL algorithms can process raw, unstructured data, including images, texts, and clinical notes, without requiring human supervision, allowing for the automatic extraction and analysis of clinically meaningful features. This makes DL particularly effective in handling unstructured clinical data such as radiographic images and electronic health records [2, 4].

As the global demand for TKA increases in line with an aging population, AI technologies, particularly ML and DL, are gaining recognition for their potential to enhance patient satisfaction, improve surgical precision, and reduce healthcare costs [7]. In recent years, AI has shown clinical utility across all stages of TKA, including patient selection and surgical indications, preoperative planning and simulation, intraoperative assistance, and postoperative monitoring and rehabilitation [8]. By leveraging large-scale clinical datasets, ML and DL algorithms enable precise patient stratification, optimize surgical plans, provide real-time feedback during procedures, and facilitate tailored postoperative recovery strategies [5].

Furthermore, DL-based AI is gaining prominence in robotic-assisted knee arthroplasty [6]. The combination of AI, robotic systems, and augmented reality (AR) technologies allows for accurate preoperative predictions of bone resections and implant sizing. Intraoperatively, AI-powered robotic systems provide real-time kinematic feedback based on individual limb alignment and soft tissue tension, thereby supporting dynamic intraoperative decision-making and enhancing surgical accuracy [6, 7]. These systems are expected to improve surgical outcomes by minimizing alignment outliers and enhancing overall patient satisfaction [8].

Patient satisfaction is a critical metric in evaluating TKA outcomes. Numerous factors, including age, sex, comorbidities, and lower limb alignment, influence postoperative satisfaction [9]. Integrating these multifactorial determinants into DL-based predictive models could enhance outcome forecasting and reduce the incidence of suboptimal results [10]. In addition, AI-assisted postoperative care tailored to individual patient profiles may enhance recovery trajectories and overall satisfaction [6].

Despite these promising developments, a clear synthesis of the evidence across the entire clinical workflow is needed to guide clinicians. Therefore, this structured review aims to explore the current applications of AI in TKA, examine how these technologies are being implemented in clinical practice, and critically assess their potential—and limitations—for improving surgical outcomes and patient care. This review is structured to follow the TKA workflow, covering patient selection, preoperative planning, intraoperative execution, and postoperative monitoring, concluding with a discussion of current challenges and future directions.

## Methods

### Literature search strategy

This study was conducted as a structured review to synthesize the clinical applications and implications of artificial intelligence in total knee arthroplasty (TKA). A comprehensive literature search was performed using the PubMed, Scopus, and Google Scholar electronic databases for articles published between January 2015 and March 2025. This timeframe was chosen to capture the recent acceleration of AI development and adoption in orthopedic surgery. The search strategy utilized a combination of Medical Subject Headings (MeSH) and free-text keywords, including: (“artificial intelligence” OR “machine learning” OR “deep learning”) AND (“total knee arthroplasty” OR “knee replacement”) AND (“robotics” OR “augmented reality” OR “virtual reality” OR “preoperative planning” OR “postoperative monitoring” OR “patient selection” OR “surgical simulation”).

### Inclusion and exclusion criteria

Studies were included if they met the following criteria: (1) published in the English language; (2) available in full-text format; (3) focused on the application of AI, ML, or DL technologies at any stage of the TKA workflow (preoperative, intraoperative, or postoperative); and (4) were original clinical studies, review articles, or meta-analyses.

Exclusion criteria were: (1) articles not published in English; (2) abstracts, conference proceedings, or editorials without sufficient data; (3) studies where the primary focus was not TKA; (4) case reports; and (5) preclinical or animal studies. The initial search results were screened by title and abstract, and relevant full-text articles were subsequently retrieved and assessed for eligibility.

### Data extraction and synthesis

Data from the selected articles were extracted and organized thematically according to the phases of the TKA care continuum: patient selection and surgical indication, preoperative planning, intraoperative decision support, and postoperative assessment. Owing to the heterogeneity of the included studies and technologies, a quantitative meta-analysis was not performed. Instead, a synthesis was conducted to summarize key findings, identify trends, and discuss the clinical implications and limitations of the current evidence.

## Artificial intelligence for patient selection and surgical indication decision

Numerous studies have emphasized that one of the most critical factors influencing the success of TKA is the appropriate selection of surgical candidates [5, 11]. Traditionally, orthopedic surgeons have relied on a combination of clinical assessments, including the patient’s pain level, functional limitations, and radiographic severity of osteoarthritis, often evaluated using the Kellgren–Lawrence (K–L) grading system. However, these conventional methods are inherently subjective and prone to interobserver variability, limiting their consistency and reproducibility across clinical settings.

To address these challenges, recent advances in ML have led to the development of algorithms capable of integrating diverse datasets, including patient-reported outcome measures (PROMs), demographic characteristics (such as age, sex, and body mass index [BMI]), and medical comorbidities, to generate objective, data-driven assessments of surgical necessity [12]. These predictive models are increasingly being employed to support individualized decision-making regarding TKA indications, potentially reducing clinician bias and improving the precision of patient selection.

DL models, in particular, have shown promise in automating the evaluation of radiographic features such as the K–L grading. This reduces interobserver discrepancies and enables more consistent classification of osteoarthritis severity [6, 13]. Furthermore, DL algorithms can identify critical preoperative variables that correlate with postoperative complications. For instance, Jo et al. [14] developed a gradient boosting machine model that predicted the need for transfusion after TKA with good performance (AUC 0.842) using only six preoperative variables, including hemoglobin level and patient age. Similarly, Yeo et al. [15] used multiple ML models to predict surgical site infections, achieving an AUC of up to 0.84 with an artificial neural network (ANN). However, a systematic review by Karlin et al. [5] noted that while many such models exist, only a small fraction (9 out of 30 reviewed studies) have been externally validated, highlighting a critical gap between development and clinical readiness.

Beyond primary TKA, AI-based systems are also being explored in revision knee arthroplasty. These systems can assist in diagnosing implant loosening and identifying appropriate implant models through radiographic pattern recognition [3, 7]. Thus, AI offers a complementary approach to traditional guideline-based decision-making by enabling the personalization of surgical thresholds and optimizing the indications for surgical intervention [16, 17].

Moreover, AI and ML techniques are being increasingly applied to predict postoperative recovery trajectories and patient satisfaction, two critical outcomes that significantly influence the overall treatment success [2, 7]. With the growing availability of large-scale electronic health records and structured datasets, the development of robust predictive models for TKA outcomes is becoming more feasible [9]. These models are designed to incorporate a wide range of factors, including patient characteristics, preoperative pain levels, comorbidities, psychological well-being, and socioeconomic indicators, to better evaluate their collective influence on recovery and satisfaction post-surgery [5, 11].

Key predictors of postoperative outcomes include baseline pain scores (such as the visual analog scale), joint-specific clinical scores (such as the Knee Injury and Osteoarthritis Outcome Score and Western Ontario and McMaster Universities Osteoarthritis Index), range of motion, and quality-of-life-related PROMs such as the EuroQol 5 Dimensions [6, 11]. Measures of mental health, including anxiety, depression, and general health status (12-item Short Form Health Survey), are also being integrated into prediction models. In addition, clinical and demographic factors such as the American Society of Anesthesiologists physical status classification, BMI, sex, age, history of prior knee surgeries, severity of radiographic arthritis, and preoperative limb alignment are being evaluated as significant predictors [8].

Despite these advancements, most existing models remain at developmental or preclinical stages [6]. While early results have been promising, there is still a lack of validated and widely applicable models suitable for seamless integration into routine clinical practice [9]. Further research is needed to refine these models, validate their performance across diverse populations, and assess their real-world impact on clinical outcomes and decision-making processes.

## Artificial intelligence-assisted preoperative planning and simulation

Preoperative planning is a critical step that significantly influences TKA outcome [18]. Traditionally, surgeons have relied on manual templating using two-dimensional radiographs to estimate implant size and alignment. However, this method is inherently limited by operator-dependent variability and lacks precision, particularly in anatomically complex cases [7]. The accuracy of such conventional approaches often depends on the surgeon’s experience, which can lead to inconsistent outcomes.

Recent advances in AI, particularly in DL-based imaging technologies, have ushered in a new era of precision and personalization in preoperative planning. DL algorithms utilizing three-dimensional (3D) segmentation techniques can now accurately extract bone morphology from computed tomography (CT) scans or even plain radiographs [19]. These models enable detailed anatomical reconstruction and individualized implant planning tailored to each patient’s specific anatomy. Notably, AI systems have demonstrated high accuracy in predicting optimal implant size. A recent systematic review by Salman et al. [12] found that AI models achieved an accuracy of 88.3–99.7% for femoral components and 90–99.9% for tibial components within a ± 1 size deviation. In a direct comparative study, Lan et al. [20] reported that an AI-based 3D planning tool achieved 90% accuracy for exact femoral size prediction, significantly outperforming 2D templating (66.7%). This increased precision in preoperative planning is crucial, as an AI-based plan was shown by Lambrechts et al. [18] to reduce the number of surgeon corrections by 39.7% compared with a standard manufacturer’s plan, potentially saving time and reducing intraoperative variability. This marks a significant advancement in reducing interobserver variability and subjectivity, which have historically hindered preoperative planning [21].

AI-assisted planning tools enhance surgical precision by supporting the selection of implants that best fit a patient’s unique anatomy. They facilitate optimized alignment strategies, which are critical for the long-term success of implants [10]. Furthermore, these systems can help standardize the planning process across varying levels of surgical experience, thereby contributing to more consistent and reproducible surgical outcomes. Nevertheless, some limitations remain. Current predictive models are often based on a narrow set of input variables, and their recommendations may be limited to implant options from specific manufacturers or product lines [5]. Therefore, broader data integration and device-agnostic algorithm development are essential to maximize clinical applicability.

In parallel with advancements in planning, AI is also revolutionizing surgical training and simulation. The emergence of DL-powered immersive virtual reality (VR) platforms is transforming how orthopedic surgeons are educated and prepared for complex procedures [3, 6]. These AI-driven training systems offer real-time guidance during simulated surgeries, allowing users to make informed decisions regarding implant positioning, orientation, and instrumentation. Simulation environments can track technical errors, measure procedural efficiency, and offer performance feedback, thus enhancing the surgeon’s ability to minimize operative time and optimize intraoperative tool use.

This immersive simulation not only accelerates skill acquisition for trainees but also provides a risk-free environment for experienced surgeons to refine techniques or rehearse complex cases.

In summary, the integration of AI and DL technologies into preoperative planning and surgical simulation holds great promise for enhancing surgical accuracy, reducing variability, and improving clinical outcomes in TKA. As these technologies continue to evolve, they are expected to bridge the gap between surgical intuition and data-driven precision, ultimately transforming preoperative workflows into more standardized and predictive processes.

## Artificial intelligence for intraoperative decision support and sensing

Real-time intraoperative decision-making plays a crucial role in determining the precision, consistency, and overall success of TKA. Intraoperative variability, particularly in balancing soft tissues and achieving accurate alignment, can significantly affect postoperative stability and long-term patient outcomes. To address this problem, AI technologies are increasingly being incorporated into intraoperative workflows, providing surgeons with data-driven feedback and enhanced surgical control.

AI now contributes to intraoperative navigation, robotic arm control, and dynamic soft tissue balancing, offering real-time decision support that augments surgical accuracy [5]. A particularly notable advancement is the integration of load-sensing technology with AI. Verstraete et al. [22] demonstrated that an ML algorithm could use intraoperative load data to guide surgical corrections, achieving a high accuracy (AUC 0.89). More recently, Al-Nasser et al. [19] developed a novel AI-based sensor that could predict both load and its location across the entire tibial surface—not just within a confined triangular area—with an average accuracy of 83.4% for load and 84.6% for location. While these tools offer objective feedback to aid surgeons in achieving ligament balance, a major cause for early TKA revisions, their direct impact on long-term patient outcomes requires further validation through prospective clinical trials. These systems analyze intraoperative load data to help surgeons achieve balanced ligamentous structures, thereby minimizing the risk of postoperative instability, malalignment, and implant failure.

Moreover, DL-based AI is evolving rapidly alongside robotic surgery and AR platforms. In robotic-assisted knee arthroplasty, AI facilitates the real-time analysis of intraoperative kinematic data, providing feedback on cutting angles, gap measurements, and joints [23–25]. This feedback allows surgeons to perform personalized procedures with high precision and reproducibility, guided by preoperative plans that dynamically adapt to intraoperative findings. Consequently, AI-equipped robotic systems are becoming increasingly popular in both academic and community orthopedic settings.

Despite growing adoption, current robotic platforms remain semi-autonomous, relying heavily on human oversight and lacking the cognitive capabilities to make independent surgical decisions [7]. DL has the potential to bridge this gap by enabling robotic systems to better interpret complex anatomical structures, predict surgical outcomes, and intelligently respond to intraoperative changes [2]. Enhanced perception and autonomous adaptation through AI may ultimately lead to more intelligent and efficient robotic systems capable of higher levels of surgical autonomy.

Beyond robotics, AR-based navigation systems have emerged as promising tools for intraoperative visualization and decision support. AR technology overlays clinical information directly onto the surgical field using a combination of tracking, computing, and visualization. Unlike bulky robotic systems, AR devices are typically more compact, cost-effective, and easier to integrate into existing surgical workflows [9]. AR systems allow surgeons to visualize anatomical landmarks and implant alignment in real time, potentially improving surgical accuracy without significantly increasing operative time or complexity.

Recent commercial systems, such as Pixee Medical and NextAR^™^, represent the latest innovations in AR-based navigation. Pixee Medical employs QR code markers and smart glasses to generate intraoperative 3D coordinate systems, aiding real-time alignment and positioning [24]. NextAR^™^ integrates preoperative CT imaging with real-time soft tissue feedback using ligament-balancing sensors [26]. These systems offer novel capabilities; however, their clinical validations remain limited. Questions persist regarding their long-term accuracy, reproducibility, and cost-effectiveness compared with conventional and robotic techniques [10].

Further research is necessary to determine whether AR-based navigation systems can demonstrably improve patient outcomes and offer practical value in routine surgical practice. Nonetheless, the future integration of AR with AI-powered analytics and feedback systems holds significant potential. Together, these technologies may enhance surgical planning and execution, contributing to more precise, efficient, and patient-specific procedures [6]. As they continue to evolve, they are expected to redefine the intraoperative environment and transform surgical decision-making into a more intelligent and responsive process.

## Postoperative assessment and monitoring

Postoperative monitoring is essential for evaluating recovery progress and detecting early signs of complications post-TKA. As recovery trajectories can vary significantly between patients, timely identification of deviations is critical to prevent adverse outcomes. In this context, DL-based AI systems are increasingly being explored for their potential to enhance postoperative monitoring.

Recent developments in remote monitoring technologies have enabled the continuous collection of both subjective and objective patient data using commonly available devices, such as smartphones and wearable sensors. Early iterations of these technologies faced several limitations, including poor interoperability between applications, low user compliance, and the high cost of external sensors [6]. However, technological advancements have led to more streamlined systems that integrate wearable devices, such as smartwatches and inertial sensors, for real-time gait analysis as well as mobile applications designed to collect PROMs [2, 10]. These improvements have made remote monitoring more accessible and clinically feasible, even in settings beyond specialized research. Persona IQ^®^, developed by Zimmer Biomet, is a tibial implant with an integrated stem-based sensor that automatically collects gait and range of motion data directly from the knee. This information is transmitted to the mymobility^®^ Care Management platform, enabling care teams to monitor postoperative recovery and deliver personalized rehabilitation, with the goal of improving patient outcomes and clinical efficiency.

**Fig. 1:**
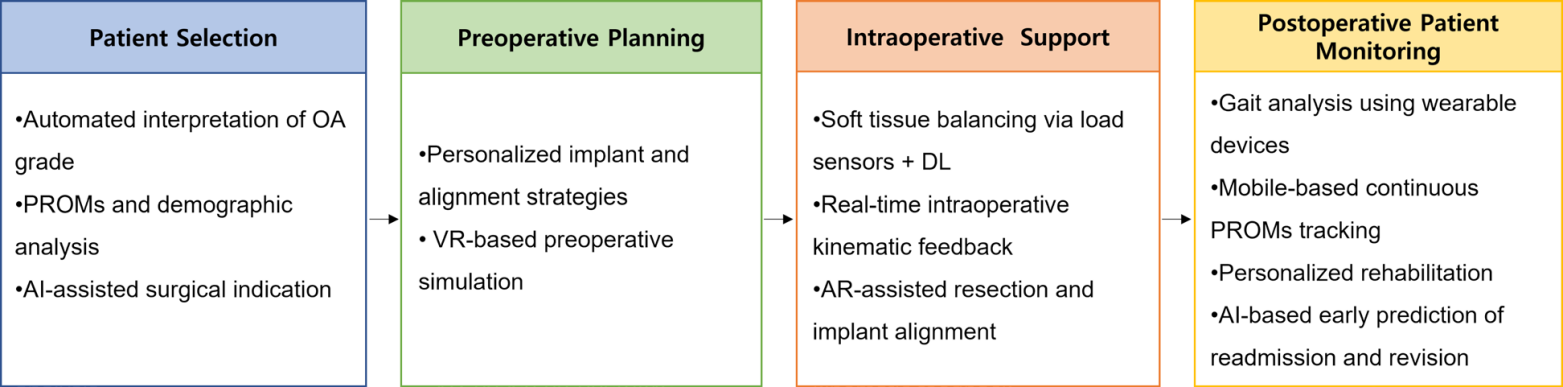
Workflow of artificial intelligence integration in total knee arthroplasty. *OA* osteoarthritis, *PROMs* patient-reported outcome measures, *AI* artificial intelligence, *VR* virtual reality, *AR* augmented reality, *DL* deep learning

Using these systems, patients can actively engage in physical therapy and home exercise programs in real time using their smartphones. Clinicians, in turn, can remotely monitor patients’ rehabilitation progress and receive alerts when individuals do not meet expected recovery milestones [6]. When deviations are identified, healthcare providers can intervene through teleconsultation or in-person follow-ups, enabling a more proactive and individualized approach to postoperative care (Fig. 1).

A recent pilot study involving 25 patients undergoing TKA reported the feasibility of continuous, passive data collection using smartphone-based monitoring platforms [27]. The findings in this study showed that such systems could effectively capture data without requiring active patient input, thereby minimizing patient burden and enhancing compliance. A randomized clinical trial by Mehta et al. [28] involving 242 patients who underwent hip or knee replacement reported a statistically significant reduction in rehospitalization rates in the group using a remote patient monitoring (RPM) system compared with the standard care group. These findings underscore the clinical utility of AI-supported remote monitoring for improving postoperative outcomes.

AI plays a pivotal role in these systems by integrating and analyzing multivariate data to provide actionable insights. By learning from large-scale datasets, AI algorithms can identify patients at risk of delayed recovery, implant-related complications, or suboptimal functional outcomes. In addition, these systems can help generate personalized rehabilitation pathways on the basis of real-time performance data, ensuring that care plans are tailored to each patient’s needs.

Furthermore, ongoing research is exploring the integration of national joint registry data with AI-driven prognostic modeling. These combined datasets enable the prediction of key clinical endpoints, such as 90-day readmission risk, revision surgery probability, and long-term functional recovery trajectories [29]. As the volume and quality of clinical data continue to grow, these models are expected to become increasingly accurate and clinically valuable, providing healthcare providers with robust tools for early intervention and outcome optimization.

A concise summary of the included AI applications, key studies, reported outcomes, and levels of evidence is presented in Table 1.

**Table 1:** Summary of AI applications by TKA phase

TKA phase	AI application	Key studies	Key findings/reported outcomes	Level of evidence/limitations
Patient selection	Complication prediction	Jo et al. [14], Yeo et al. [15]	- Developed a model to predict postoperative transfusion (AUC: 0.842) - Developed a model to predict surgical site infection (SSI) (AUC: up to 0.84)	- Validity of specific prediction models demonstrated - However, as noted by Karlin et al. [5], only a few models have been externally validated, limiting clinical applicability
Preoperative planning	Implant size prediction	Salman et al. [12], Lan et al. [20]	- AI models achieved high prediction accuracy within ± 1 size deviation: 88.3–99.7% for femoral and 90–99.9% for tibial components - AI-based 3D planning was superior to 2D templating for exact size prediction (femoral: 90% versus 66.7%)	- High accuracy confirmed by systematic review and comparative study - Limitation that models may be specific to certain manufacturers
	Improved planning efficiency	Lambrechts et al. [18]	- AI-generated surgical plans reduced the number of surgeon corrections by 39.7% compared with default plans	- Retrospective study - Considered only static bone anatomy; did not include soft-tissue data
Intraoperative support	Robotic surgery	Batailler et al. [1]	- Robotic assistance improved accuracy of knee alignment, implant positioning, and ligament balancing	- Literature review
	Ligament balancing sensor	Verstraete et al. [22], Al-Nasser et al. [19]	- ML used intraoperative load data to guide surgical corrections (AUC: 0.89) - A novel AI sensor predicted load (83.4% accuracy) and location (84.6% accuracy) across the entire tibial surface	- Demonstrates potential to improve surgical accuracy with objective data - Impact on long-term patient outcomes requires validation in prospective studies
Postoperative monitoring	Remote patient monitoring (RPM)	Mehta et al. [28], Ramkumar et al. [27]	- Statistically significant reduction in rehospitalization rates in the RPM group - Feasibility of uninterrupted data collection via wearables demonstrated (pilot study)	- Clinical utility confirmed via randomized controlled trial (RCT) - Many studies are still in pilot or small-scale phases, requiring larger validation
	Radiographic analysis	Pagano et al. [21]	- AI software analyzed radiographs more than twice as fast as experts (*p* < 0.001) - However, accuracy decreased in patients with BMI > 30 and for certain metrics (JLCA); failed to analyze 22.5% of images	- Potential for improved workflow efficiency - Performance degradation and failure rates exist under specific conditions (e.g., obesity and image quality)
Overall assessment	ML model performance	Karlin et al. [5]	- Less than 35% of predictive outcomes in reviewed studies showed “excellent” or “outstanding” AUCs - Only 9 of 30 studies outperformed traditional statistics (logistic regression)	- Systematic review - Emphasizes the need for critical assessment and validation before clinical adoption

## Current limitations and future directions

AI and DL technologies have introduced significant advancements in orthopedic surgery, particularly in TKA; however, several limitations remain that must be addressed to enable broader and more effective clinical implementation [30]. The challenges span from data quality and algorithmic bias to ethical considerations and practical barriers to deployment.

### Data quality and algorithmic bias

The performance of any AI model is fundamentally dependent on the quality and diversity of its training data. Models developed using datasets that lack representation across different ethnicities, sexes, and socioeconomic backgrounds may exhibit algorithmic bias, leading to reduced generalizability and potentially inequitable outcomes when applied in real-world clinical settings [31]. For example, systematic biases in data collection have led to the under-representation of women and racial minorities in clinical trial and registry populations. Furthermore, crucial information such as social determinants of health is often missing from electronic health records, which can hamper a model’s predictive performance and fairness.

### The “black box” problem and clinical trust

Many current AI systems function as “black boxes,” offering predictions without a clear explanation of their decision-making process. This lack of transparency limits the interpretability and clinical trustworthiness of AI, as clinicians may be hesitant to act on recommendations they do not understand. For clinicians to regularly use AI algorithms, they need to trust them. Therefore, future advancements in explainable AI (XAI) are crucial. Techniques that can highlight which input features most influenced a prediction will enhance transparency, support informed clinical judgment, and ultimately facilitate the integration of AI into routine workflows [3].

### Ethical, regulatory, and governance challenges

The use of AI in medicine introduces complex ethical and regulatory hurdles. While data privacy and patient confidentiality are paramount concerns, questions regarding data ownership also remain unanswered [1]. For instance, data collected by robotic platforms are sometimes used for product development, occasionally without the patient’s express consent for that specific purpose. Furthermore, regulatory frameworks for AI-powered medical devices must be strengthened. In the USA and Europe, bodies such as the Food and Drug Administration (FDA) and Conformité Européenne (CE) provide oversight, but clear guidelines for the validation, approval, and post-market surveillance of adaptive “learning” algorithms are still evolving. The complex legal issues related to the clinical use of an algorithm are a significant barrier to widespread implementation [1, 3, 32].

### Barriers to clinical implementation and equity

Despite thousands of AI algorithms being published, very few are successfully integrated into routine clinical care. A primary reason is the failure to generalize well outside of the training data; a systematic review found that only 30% of reviewed AI studies in arthroplasty provided validation against an external dataset. Safely deploying, monitoring, and updating a clinical AI model also requires significant technical infrastructure, a field known as MLOps, which is still new to many healthcare organizations. Finally, the high cost of advanced technologies such as robotics and AI-powered software raises concerns about equitable deployment, as it could widen the gap in care quality between well-resourced and under-resourced institutions [3, 8].

### Future directions and limitations of this review

To overcome these challenges, future efforts must prioritize the development of large-scale, multicenter datasets that reflect diverse populations. As noted by Mickley et al. [3], the next wave of AI research will likely focus on clinical implementation science, explainable AI, and the increased use of generative AI, such as large language models (LLMs).

Continuous refinement of AI models based on real-world evidence, gathered from actual clinical deployments, is also essential. Unlike controlled research environments, real-world settings present challenges such as data variability, patient noncompliance, and logistical complexities. Incorporating these factors will help ensure reliable AI performance.

Finally, we must acknowledge the limitations of this review. As a structured review, it provides a broad overview of the field but does not include a meta-analysis or a quantitative synthesis of the comparative performance of different AI models. The conclusions are therefore based on a qualitative synthesis of the available literature. By addressing the current limitations and investing in robust, ethical, and equitable infrastructure, the orthopedic community can responsibly harness AI’s capabilities to improve surgical precision, enhance patient outcomes, and elevate the standard of care.

## Conclusions

The integration of AI into the TKA workflow has the potential to drive a paradigm shift toward more data-driven, patient-specific orthopedic care. As this review has shown, AI applications are demonstrating tangible benefits, from improving the accuracy of preoperative planning and implant sizing to enabling personalized postoperative monitoring that may reduce rehospitalization rates [18, 20, 28]. These technologies offer a pathway to augment surgical precision and standardize care by transforming subjective assessments into objective, quantifiable metrics.

However, a critical appraisal of the current literature reveals that the journey from algorithmic development to widespread, reliable clinical adoption is fraught with significant challenges. Despite promising results in controlled settings, a crucial gap remains in robust, multicenter external validation; indeed, some reviews indicate that less than a third of published models have been validated on external datasets [5]. Issues of algorithmic bias, the “black box” nature of complex models, and unresolved ethical and regulatory hurdles surrounding data governance and device approval present formidable barriers to implementation [3].

Therefore, while the promise of AI in TKA is substantial, its future success hinges on a commitment to responsible innovation. The orthopedic community must embrace AI not as a replacement for clinical expertise but as a complementary tool that requires rigorous scrutiny. Safe, effective, and equitable integration will demand ongoing interdisciplinary collaboration, the development of explainable and transparent algorithms, and—most importantly—the accumulation of high-quality clinical evidence to prove not only the validity and utility of these technologies but also their cost-effectiveness in real-world practice.

## Data Availability

Not applicable.

## Abbreviations

AI: Artificial Intelligence
DL: Deep Learning
ML: Machine Learning
TKA: Total knee arthroplasty
PROMs: Patient-reported outcome measures
K-L grading: Kellgren–Lawrence grading
CT: Computed tomography
AR: Augmented reality
VR: Virtual reality
3D: Three-dimensional

## References

[1] Batailler C, Shatrov J, Sappey-Marinier E, Servien E, Parratte S, Lustig S (2022) Artificial intelligence in knee arthroplasty: current concept of the available clinical applications. Arthroplasty 4(1):17. 10.1186/s42836-022-00119-6 35491420 10.1186/s42836-022-00119-6 PMC9059406

[2] Helm JM, Swiergosz AM, Haeberle HS, Karnuta JM, Schaffer JL, Krebs VE et al (2020) Machine learning and artificial intelligence: definitions, applications, and future directions. Curr Rev Musculoskelet Med 13(1):69–76. 10.1007/s12178-020-09600-8 31983042 10.1007/s12178-020-09600-8 PMC7083992

[3] Mickley JP, Kaji ES, Khosravi B, Mulford KL, Taunton MJ, Wyles CC (2024) Overview of artificial intelligence research within hip and knee arthroplasty. Arthroplasty Today 27:101396. 10.1016/j.artd.2024.101396 39071822 10.1016/j.artd.2024.101396 PMC11282426

[4] Yamashita R, Nishio M, Do RKG, Togashi K (2018) Convolutional neural networks: an overview and application in radiology. Insights Imaging 9(4):611–629. 10.1007/s13244-018-0639-9 29934920 10.1007/s13244-018-0639-9 PMC6108980

[5] Karlin EA, Lin CC, Meftah M, Slover JD, Schwarzkopf R (2023) The impact of machine learning on total joint arthroplasty patient outcomes: a systemic review. J Arthroplasty 38(10):2085–2095. 10.1016/j.arth.2022.10.039 36441039 10.1016/j.arth.2022.10.039

[6] Liu X, Li S, Zou X, Chen X, Xu H, Yu Y et al (2024) Development and clinical validation of a deep learning-based knee CT image segmentation method for robotic-assisted total knee arthroplasty. Int J Med Robot 20(4):e2664. 10.1002/rcs.2664 38994900 10.1002/rcs.2664

[7] Longo UG, De Salvatore S, Valente F, Villa Corta M, Violante B, Samuelsson K (2024) Artificial intelligence in total and unicompartmental knee arthroplasty. BMC Musculoskelet Disord 25(1):571. 10.1186/s12891-024-07516-9 39034416 10.1186/s12891-024-07516-9 PMC11265144

[8] Myers TG, Ramkumar PN, Ricciardi BF, Urish KL, Kipper J, Ketonis C (2020) Artificial intelligence and orthopaedics: an introduction for clinicians. J Bone Joint Surg Am 102(9):830–840. 10.2106/jbjs.19.01128 32379124 10.2106/JBJS.19.01128 PMC7508289

[9] Pailhé R (2021) Total knee arthroplasty: latest robotics implantation techniques. Orthop Traumatol Surg Res 107(1s):102780. 10.1016/j.otsr.2020.102780 33333275 10.1016/j.otsr.2020.102780

[10] Panchmatia JR, Visenio MR, Panch T (2018) The role of artificial intelligence in orthopaedic surgery. Br J Hosp Med (Lond) 79(12):676–681. 10.12968/hmed.2018.79.12.676 30526106 10.12968/hmed.2018.79.12.676

[11] Bourne RB, Chesworth BM, Davis AM, Mahomed NN, Charron KD (2010) Patient satisfaction after total knee arthroplasty: who is satisfied and who is not? Clin Orthop Relat Res 468(1):57–63. 10.1007/s11999-009-1119-9 19844772 10.1007/s11999-009-1119-9 PMC2795819

[12] Salman LA, Khatkar H, Al-Ani A, Alzobi OZ, Abudalou A, Hatnouly AT et al (2024) Reliability of artificial intelligence in predicting total knee arthroplasty component sizes: a systematic review. Eur J Orthop Surg Traumatol 34(2):747–756. 10.1007/s00590-023-03784-8 38010443 10.1007/s00590-023-03784-8 PMC10858112

[13] Lee DW, Song DS, Han H-S, Ro DH (2024) Accurate, automated classification of radiographic knee osteoarthritis severity using a novel method of deep learning: plug-in modules. Knee Surg Rel Res 36(1):24. 10.1186/s43019-024-00228-3 10.1186/s43019-024-00228-3 PMC11323666 39138550

[14] Jo C, Ko S, Shin WC, Han HS, Lee MC, Ko T et al (2020) Transfusion after total knee arthroplasty can be predicted using the machine learning algorithm. Knee Surg Sports Traumatol Arthrosc 28(6):1757–1764. 10.1007/s00167-019-05602-3 31254027 10.1007/s00167-019-05602-3

[15] Yeo I, Klemt C, Robinson MG, Esposito JG, Uzosike AC, Kwon Y-M (2023) The use of artificial neural networks for the prediction of surgical site infection following TKA. J Knee Surg 36(06):637–643 35016246 10.1055/s-0041-1741396

[16] Rodríguez-Merchán EC (2022) The current role of the virtual elements of artificial intelligence in total knee arthroplasty. EFORT Open Rev 7(7):491–497. 10.1530/eor-21-0107 35900206 10.1530/EOR-21-0107 PMC9297054

[17] Kwon SB, Ku Y, Han HS, Lee MC, Kim HC, Ro DH (2020) A machine learning-based diagnostic model associated with knee osteoarthritis severity. Sci Rep 10(1):15743. 10.1038/s41598-020-72941-4 32978506 10.1038/s41598-020-72941-4 PMC7519044

[18] Lambrechts A, Wirix-Speetjens R, Maes F, Van Huffel S (2022) Artificial intelligence based patient-specific preoperative planning algorithm for total knee arthroplasty. Front Robot AI 9:840282. 10.3389/frobt.2022.840282 35350703 10.3389/frobt.2022.840282 PMC8957999

[19] Al-Nasser S, Noroozi S, Harvey A, Aslani N, Haratian R (2024) Exploring the performance of an artificial intelligence-based load sensor for total knee replacements. Sensors (Basel). 10.3390/s24020585 38257676 10.3390/s24020585 PMC10821047

[20] Lan Q, Li S, Zhang J, Guo H, Yan L, Tang F (2024) Reliable prediction of implant size and axial alignment in AI-based 3D preoperative planning for total knee arthroplasty. Sci Rep 14(1):16971. 10.1038/s41598-024-67276-3 39043748 10.1038/s41598-024-67276-3 PMC11266554

[21] Pagano S, Müller K, Götz J, Reinhard J, Schindler M, Grifka J et al (2023) The role and efficiency of an AI-powered software in the evaluation of lower limb radiographs before and after total knee arthroplasty. J Clin Med. 10.3390/jcm12175498 37685563 10.3390/jcm12175498 PMC10487842

[22] Verstraete MA, Moore RE, Roche M, Conditt MA (2020) The application of machine learning to balance a total knee arthroplasty. Bone Joint Open 1(6):236–244. 10.1302/2633-1462.16.Bjo-2020-0056.R1 33225295 10.1302/2633-1462.16.BJO-2020-0056.R1 PMC7677727

[23] Sabatini L, Bosco F, Barberis L, Camazzola D, Bistolfi A, Risitano S et al (2021) Kinetic sensors for ligament balance and kinematic evaluation in anatomic bi-cruciate stabilized total knee arthroplasty. Sensors. 10.3390/s21165427 34450869 10.3390/s21165427 PMC8399549

[24] Lambrechts J, Vansintjan P, Lapierre C, Sinnaeve F, Van Lysebettens W, Van Overschelde P (2024) Accuracy of a new augmented reality assisted technique for total knee arthroplasty: an *in vivo* study. Arthroplasty Today 30:101565. 10.1016/j.artd.2024.101565 39524992 10.1016/j.artd.2024.101565 PMC11550726

[25] Iyengar KP, Gowers BTV, Jain VK, Ahluwalia RS, Botchu R, Vaishya R (2021) Smart sensor implant technology in total knee arthroplasty. J Clin Orthop Trauma 22:101605. 10.1016/j.jcot.2021.101605 34631412 10.1016/j.jcot.2021.101605 PMC8479248

[26] Sabatini L, Ascani D, Vezza D, Masse A, Cacciola G (2024) Novel surgical technique for total knee arthroplasty integrating kinematic alignment and real-time elongation of the ligaments using the NextAR system. J Pers Med. 10.3390/jpm14080794 39201986 10.3390/jpm14080794 PMC11355594

[27] Ramkumar PN, Haeberle HS, Ramanathan D, Cantrell WA, Navarro SM, Mont MA et al (2019) Remote patient monitoring using mobile health for total knee arthroplasty: validation of a wearable and machine learning-based surveillance platform. J Arthroplasty 34(10):2253–2259. 10.1016/j.arth.2019.05.021 31128890 10.1016/j.arth.2019.05.021

[28] Mehta SJ, Hume E, Troxel AB, Reitz C, Norton L, Lacko H et al (2020) Effect of remote monitoring on discharge to home, return to activity, and rehospitalization after hip and knee arthroplasty: a randomized clinical trial. JAMA Netw Open 3(12):e2028328. 10.1001/jamanetworkopen.2020.28328 33346847 10.1001/jamanetworkopen.2020.28328 PMC7753899

[29] Park J, Zhong X, Miley EN, Rutledge RS, Kakalecik J, Johnson MC et al (2024) Machine learning-based predictive models for 90-day readmission of total joint arthroplasty using comprehensive electronic health records and patient-reported outcome measures. Arthroplasty Today 25:101308. 10.1016/j.artd.2023.101308 38229870 10.1016/j.artd.2023.101308 PMC10790030

[30] Topol EJ (2019) High-performance medicine: the convergence of human and artificial intelligence. Nat Med 25(1):44–56. 10.1038/s41591-018-0300-7 30617339 10.1038/s41591-018-0300-7

[31] Esteva A, Robicquet A, Ramsundar B, Kuleshov V, DePristo M, Chou K et al (2019) A guide to deep learning in healthcare. Nat Med 25(1):24–29. 10.1038/s41591-018-0316-z 30617335 10.1038/s41591-018-0316-z

[32] Cigdem O, Deniz CM (2023) Artificial intelligence in knee osteoarthritis: a comprehensive review for 2022. Osteoarthritis Imaging. 10.1016/j.ostima.2023.100161 38948116 10.1016/j.ostima.2023.100161 PMC11213283

